# 3-(1*H*-Imidazol-1-yl)propanaminium 2-carb­oxy-4,6-di­nitro­phenolate

**DOI:** 10.1107/S1600536814003146

**Published:** 2014-02-19

**Authors:** Thammarse S. Yamuna, Manpreet Kaur, Brian J. Anderson, Jerry P. Jasinski, H.S. Yathirajan

**Affiliations:** a Department of Studies in Chemistry, University of Mysore, Manasagangotri, Mysore 570 006, India; b Department of Chemistry, Keene State College, 229 Main Street, Keene, NH 03435-2001, USA

## Abstract

In the title salt, C_6_H_12_N_3_^+^·C_7_H_3_N_2_O_7_^−^, the imidazole ring is planar, with a maximum deviation of 0.0013 (14) Å for the N attached to the propanaminium group. In the anion, a single intra­molecular O—H⋯O hydrogen bond is observed. The mean planes of the nitro groups in the anion are twisted from the benzene ring mean plane making dihedral angles of 24.7 (9) and 3.9 (6)°. In the crystal, the ammonium H atoms form N—H⋯N and N—H⋯O hydrogen bonds, resulting in an infinite chain along [111]. In addition to the classical hydrogen bonds, weak C—H⋯O and π–π [centroid–centroid distance = 3.7124 (9) Å] inter­actions are also observed, which lead to the formation a three-dimensional supramolecular structure that links the chains into layers along the *bc* plane.

## Related literature   

For general background and the pharmacological properties of imidazole compounds, see: ten Have *et al.* (1997 ▶); Lombardino & Wiseman (1974 ▶); Jackson *et al.* (2000 ▶); Krezel (1998 ▶); Maier *et al.* (1989 ▶). For the related structures of substituted imidazoles, see: Dayananda *et al.* (2012 ▶); Hemamalini & Fun (2010 ▶); Jasinski *et al.* (2011 ▶); Wei *et al.* (2012 ▶); Yamuna *et al.* (2013 ▶).
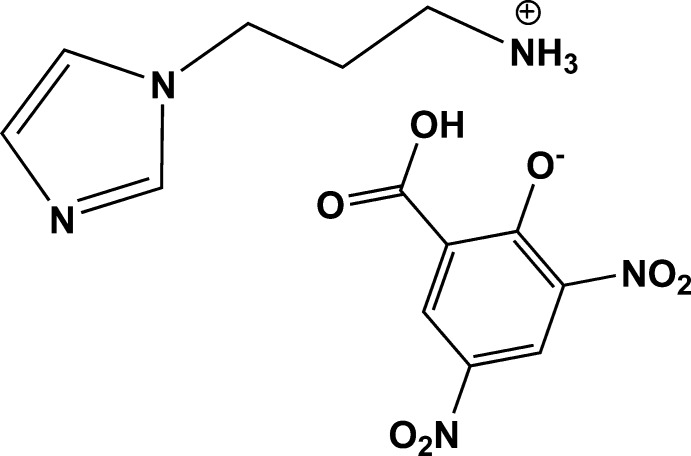


## Experimental   

### 

#### Crystal data  

C_6_H_12_N_3_^+^·C_7_H_3_N_2_O_7_^−^
*M* *_r_* = 353.30
Triclinic, 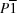

*a* = 7.0109 (4) Å
*b* = 10.6617 (8) Å
*c* = 10.7454 (7) Å
α = 93.075 (6)°
β = 95.863 (5)°
γ = 104.944 (6)°
*V* = 769.30 (9) Å^3^
*Z* = 2
Cu *K*α radiation
μ = 1.09 mm^−1^
*T* = 173 K
0.22 × 0.14 × 0.12 mm

#### Data collection  

Agilent Xcalibur (Eos, Gemini) diffractometer
Absorption correction: multi-scan (*CrysAlis PRO* and *CrysAlis RED*; Agilent, 2012 ▶) *T*_min_ = 0.925, *T*_max_ = 1.000
4664 measured reflections
2953 independent reflections
2582 reflections with *I* > 2σ(*I*)
*R*_int_ = 0.026

#### Refinement  

*R*[*F* ^2^ > 2σ(*F* ^2^)] = 0.042
*wR*(*F* ^2^) = 0.122
*S* = 1.04
2953 reflections
229 parameters
H-atom parameters constrained
Δρ_max_ = 0.25 e Å^−3^
Δρ_min_ = −0.25 e Å^−3^

### 

Data collection: *CrysAlis PRO* (Agilent, 2012 ▶); cell refinement: *CrysAlis PRO*; data reduction: *CrysAlis RED* (Agilent, 2012 ▶); program(s) used to solve structure: *SUPERFLIP* (Palatinus & Chapuis, 2007 ▶); program(s) used to refine structure: *SHELXL2012* (Sheldrick, 2008 ▶); molecular graphics: *OLEX2* (Dolomanov *et al.*, 2009 ▶); software used to prepare material for publication: *OLEX2*.

## Supplementary Material

Crystal structure: contains datablock(s) I. DOI: 10.1107/S1600536814003146/fj2659sup1.cif

Structure factors: contains datablock(s) I. DOI: 10.1107/S1600536814003146/fj2659Isup2.hkl

Supporting information file. DOI: 10.1107/S1600536814003146/fj2659Isup3.cml

CCDC reference: 986378

Additional supporting information:  crystallographic information; 3D view; checkCIF report

## Figures and Tables

**Table 1:** Hydrogen-bond geometry (Å, °)

*D*—H⋯*A*	*D*—H	H⋯*A*	*D*⋯*A*	*D*—H⋯*A*
O2*B*—H2*B*⋯O1*B*	0.84	1.66	2.4484 (15)	155
N3*A*—H3*AA*⋯N1*A* ^i^	0.91	1.92	2.7987 (19)	162
N3*A*—H3*AB*⋯O1*B* ^ii^	0.91	2.03	2.8153 (17)	144
N3*A*—H3*AC*⋯O3*B* ^iii^	0.91	2.07	2.9546 (17)	165
C4*A*—H4*AB*⋯O4*B* ^iv^	0.99	2.53	3.3572 (19)	142

Symmetry codes: (i) 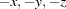; (ii) 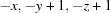; (iii) 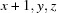; (iv) 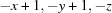.

**Fig. 1.**
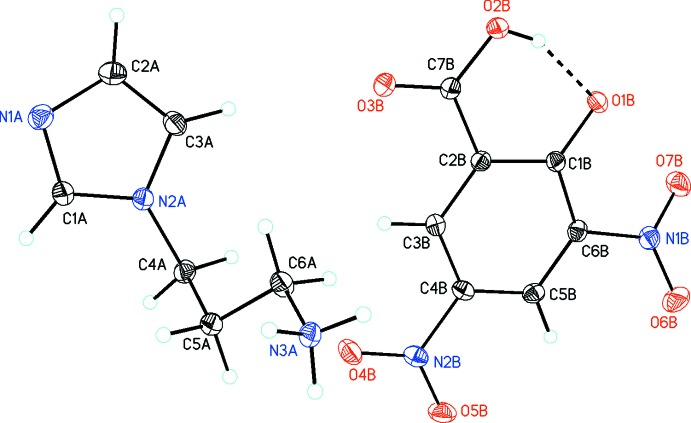
ORTEP drawing of (I) ( C6H12N3+.C7H3N2O7-) showing the labeling scheme with 30% probability displacement ellipsoids. Dashed lines indicate a O2B—H2B···O1B intramolecular hydrogen bond in the anion within the asymmetric unit.

**Fig. 2.**
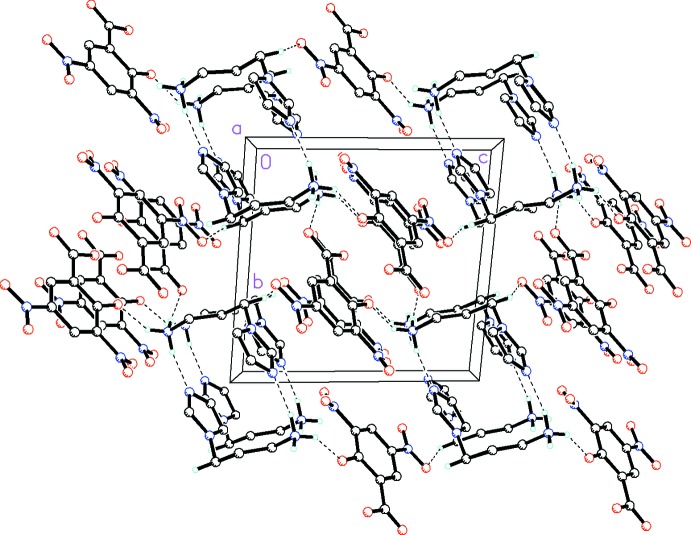
Molecular packing for (I) viewed along the a axis. Dashed lines indicate N—H···O, N—H···N intermolecular hydrogen bonds and weak C—H···O intermolecular interactions. H atoms not involved in hydrogen bonding have been removed for clarity.

## supplementary crystallographic information

### 1. Comment

Imidazole rings appear frequently in biologically active compounds, both natural and man-made (ten Have *et al.*, 1997). Compounds with an imidazole ring system have many pharmacological properties and play important roles in biochemical processes (Lombardino & Wiseman, 1974). Most of the imidazole compounds are known as inhibitors of fungicides and herbicides, plant growth regulators and therapeutic agents (Maier *et al.*, 1989), anticancer agents (Krezel, 1998) and bactericidal effects (Jackson *et al.*, 2000). The crystal structures of some related compounds, viz ; 2-amino-5-methylpyridinium 2-hydroxy-3,5-dinitrobenzoate (Hemamalini *et al.*, 2010); Cinnarizinium 3,5-dinitrosalicylate (Dayananda *et al.*, 2012); Enrofloxacinium picrate (Jasinski *et al.*, 2011); 3-(1H-imidazol-1-yl)propanaminium picrate (Yamuna *et al.*, 2013); 3,5-dimethylpyrazolium 3,5-dinitrosalicylate (Wei *et al.*, 2012), have been reported. In view of the importance of substituted imidazoles and organic acid–base adducts based on hydrogen bonding and receiving great attention in recent years, this paper reports the crystal structure of the title salt, (I), C_6_H_12_N_3_^+^.C_7_H_3_N_2_O_7_^-^.

The title salt, (I), C_6_H_12_N_3_^+^.C_7_H_3_N_2_O_7_^-^, crystallizes with one independent monocation (A) and monoanion (B) in the asymmetric unit (Fig. 1). In the cation the protonated imidazol-1-ium ring is planar (maximum deviation = 0.0013 (14)Å for N2A). In the anion, a single O—H···O intramolecular hydrogen bond is observed. Bond lengths are in normal ranges. The mean planes of the nitro groups in the anion are twisted from the phenyl ring mean plane with maximun angles of 24.7 (9)° and 3.9 (6)°, respectively. The hydrogen atoms on the terminal N atom of the cation form N—H···N and N—H···O intermolecular hydrogen bonds resulting in an infinite 1D chain along [1 1 1]. In the crystal, in addition to the classical hydrogen bonds, weak C—H···O (Table 1) and Cg1—Cg2 π—π intermolecular interactions are observed with an intercentroid distance of 3.7125 (9)Å (symmetry operation -x,1-y,-z; Cg1 and Cg2 are the centroids of the C1B–C6B and N1A/C1A/N2A/C3A/C2A rings) which contribute to crystal packing stability (Fig. 2).

### 2. Experimental

Commercially available 1-(3-aminopropyl)imidazole (0.5 g, 3.99 mmol) and 3,5 dinitrosalicylic acid (0.909 g, 3.99 mmol) were dissolved in 10 ml of methanol and stirred for 15 minutes at 308 K. X-ray quality crystals were formed on slow evaporation of methanol. (m.p.: 468- 475K).

### 3. Refinement

All of the H atoms were placed in their calculated positions and then refined using the riding model with Atom—H lengths of 0.95Å (CH); 0.99Å (CH_2_); 0.84Å (OH) or 0.91Å (NH_3_) . Isotropic displacement parameters for these atoms were set to 1.2 (CH, CH_2_, NH_3_) or 1.5 (OH) times *U*_eq_ of the parent atom. Idealised ammonium and tetrahedral OH were refined as rotating groups.

### Crystal data

**Table:**

C_6_H_12_N_3_^+^·C_7_H_3_N_2_O_7_^−^	*Z* = 2
*M**_r_* = 353.30	*F*(000) = 368
Triclinic, *P*1	*D*_x_ = 1.525 Mg m^−^^3^
*a* = 7.0109 (4) Å	Cu *K*α radiation, λ = 1.54184 Å
*b* = 10.6617 (8) Å	Cell parameters from 2218 reflections
*c* = 10.7454 (7) Å	θ = 4.2–72.3°
α = 93.075 (6)°	µ = 1.09 mm^−^^1^
β = 95.863 (5)°	*T* = 173 K
γ = 104.944 (6)°	Irregular, yellow
*V* = 769.30 (9) Å^3^	0.22 × 0.14 × 0.12 mm

### Data collection

**Table:**

Agilent Xcalibur (Eos, Gemini) diffractometer	2953 independent reflections
Radiation source: Enhance (Cu) X-ray Source	2582 reflections with *I* > 2σ(*I*)
Graphite monochromator	*R*_int_ = 0.026
Detector resolution: 16.0416 pixels mm^-1^	θ_max_ = 72.5°, θ_min_ = 4.2°
ω scans	*h* = −8→5
Absorption correction: multi-scan (*CrysAlis PRO* and *CrysAlis RED*; Agilent, 2012)	*k* = −12→13
*T*_min_ = 0.925, *T*_max_ = 1.000	*l* = −13→13
4664 measured reflections	

### Refinement

**Table:**

Refinement on *F*^2^	Hydrogen site location: inferred from neighbouring sites
Least-squares matrix: full	H-atom parameters constrained
*R*[*F*^2^ > 2σ(*F*^2^)] = 0.042	*w* = 1/[σ^2^(*F*_o_^2^) + (0.0682*P*)^2^ + 0.1101*P*] where *P* = (*F*_o_^2^ + 2*F*_c_^2^)/3
*wR*(*F*^2^) = 0.122	(Δ/σ)_max_ < 0.001
*S* = 1.04	Δρ_max_ = 0.25 e Å^−^^3^
2953 reflections	Δρ_min_ = −0.25 e Å^−^^3^
229 parameters	Extinction correction: *SHELXL2012* (Sheldrick, 2008), Fc^*^=kFc[1+0.001xFc^2^λ^3^/sin(2θ)]^-1/4^
0 restraints	Extinction coefficient: 0.0087 (12)
Primary atom site location: structure-invariant direct methods	

### Special details

**Table:**

Geometry. All esds (except the esd in the dihedral angle between two l.s. planes) are estimated using the full covariance matrix. The cell esds are taken into account individually in the estimation of esds in distances, angles and torsion angles; correlations between esds in cell parameters are only used when they are defined by crystal symmetry. An approximate (isotropic) treatment of cell esds is used for estimating esds involving l.s. planes.

### Fractional atomic coordinates and isotropic or equivalent isotropic displacement parameters (Å^2^)

**Table:**

	*x*	*y*	*z*	*U*_iso_*/*U*_eq_	
O1B	−0.19166 (16)	0.67530 (11)	0.52669 (10)	0.0288 (3)	
O2B	−0.38294 (16)	0.47146 (11)	0.40815 (11)	0.0309 (3)	
H2B	−0.3493	0.5402	0.4563	0.046*	
O3B	−0.25490 (16)	0.37708 (11)	0.26154 (11)	0.0308 (3)	
O4B	0.41267 (18)	0.58644 (12)	0.16868 (12)	0.0360 (3)	
O5B	0.59770 (17)	0.75622 (12)	0.28233 (13)	0.0378 (3)	
O6B	0.34447 (19)	0.93705 (13)	0.62652 (14)	0.0466 (4)	
O7B	0.02866 (19)	0.91669 (12)	0.61489 (13)	0.0407 (3)	
N1B	0.1720 (2)	0.88464 (13)	0.58134 (13)	0.0293 (3)	
N2B	0.43937 (19)	0.67328 (13)	0.25380 (13)	0.0277 (3)	
C1B	−0.0459 (2)	0.68012 (14)	0.46271 (13)	0.0220 (3)	
C2B	−0.0571 (2)	0.57869 (14)	0.36592 (13)	0.0216 (3)	
C3B	0.0986 (2)	0.57928 (14)	0.29803 (13)	0.0224 (3)	
H3B	0.0860	0.5126	0.2331	0.027*	
C4B	0.2742 (2)	0.67709 (15)	0.32417 (14)	0.0235 (3)	
C5B	0.2969 (2)	0.77675 (14)	0.41652 (14)	0.0242 (3)	
H5B	0.4187	0.8428	0.4339	0.029*	
C6B	0.1396 (2)	0.77858 (15)	0.48287 (14)	0.0240 (3)	
C7B	−0.2410 (2)	0.46764 (15)	0.34003 (14)	0.0240 (3)	
N1A	−0.2236 (2)	0.05132 (13)	−0.17302 (13)	0.0301 (3)	
N2A	−0.01482 (18)	0.22563 (12)	−0.06974 (12)	0.0236 (3)	
N3A	0.34673 (18)	0.20535 (12)	0.28180 (12)	0.0247 (3)	
H3AA	0.3273	0.1193	0.2584	0.030*	
H3AB	0.3097	0.2146	0.3598	0.030*	
H3AC	0.4776	0.2474	0.2829	0.030*	
C1A	−0.0393 (2)	0.12597 (15)	−0.15759 (15)	0.0267 (3)	
H1A	0.0635	0.1112	−0.2029	0.032*	
C2A	−0.3211 (2)	0.10673 (16)	−0.08994 (16)	0.0311 (4)	
H2A	−0.4575	0.0745	−0.0793	0.037*	
C3A	−0.1954 (2)	0.21366 (16)	−0.02562 (15)	0.0290 (4)	
H3A	−0.2257	0.2692	0.0372	0.035*	
C4A	0.1721 (2)	0.32486 (15)	−0.02842 (14)	0.0265 (3)	
H4AA	0.1419	0.4032	0.0094	0.032*	
H4AB	0.2423	0.3502	−0.1023	0.032*	
C5A	0.3076 (2)	0.27761 (16)	0.06655 (14)	0.0270 (3)	
H5AA	0.3236	0.1929	0.0335	0.032*	
H5AB	0.4405	0.3407	0.0792	0.032*	
C6A	0.2253 (2)	0.26209 (16)	0.19094 (14)	0.0276 (3)	
H6AA	0.2200	0.3483	0.2271	0.033*	
H6AB	0.0877	0.2051	0.1769	0.033*	

### Atomic displacement parameters (Å^2^)

**Table:**

	*U*^11^	*U*^22^	*U*^33^	*U*^12^	*U*^13^	*U*^23^
O1B	0.0263 (6)	0.0300 (6)	0.0273 (6)	0.0012 (5)	0.0097 (4)	−0.0047 (4)
O2B	0.0247 (6)	0.0304 (6)	0.0321 (6)	−0.0027 (4)	0.0082 (5)	−0.0070 (5)
O3B	0.0276 (6)	0.0282 (6)	0.0318 (6)	0.0003 (5)	0.0045 (5)	−0.0081 (5)
O4B	0.0360 (6)	0.0319 (6)	0.0414 (7)	0.0081 (5)	0.0168 (5)	−0.0040 (5)
O5B	0.0229 (6)	0.0376 (7)	0.0501 (8)	0.0011 (5)	0.0111 (5)	0.0012 (6)
O6B	0.0351 (7)	0.0402 (8)	0.0540 (8)	−0.0004 (6)	−0.0049 (6)	−0.0192 (6)
O7B	0.0393 (7)	0.0324 (7)	0.0472 (8)	0.0026 (5)	0.0160 (6)	−0.0121 (6)
N1B	0.0319 (7)	0.0233 (7)	0.0293 (7)	0.0012 (5)	0.0057 (6)	−0.0025 (5)
N2B	0.0253 (7)	0.0258 (7)	0.0339 (7)	0.0072 (5)	0.0088 (5)	0.0060 (5)
C1B	0.0230 (7)	0.0232 (7)	0.0194 (7)	0.0049 (6)	0.0033 (5)	0.0023 (6)
C2B	0.0215 (7)	0.0215 (7)	0.0207 (7)	0.0035 (6)	0.0021 (5)	0.0028 (6)
C3B	0.0255 (7)	0.0216 (7)	0.0210 (7)	0.0072 (6)	0.0043 (6)	0.0016 (5)
C4B	0.0220 (7)	0.0248 (7)	0.0258 (7)	0.0076 (6)	0.0066 (6)	0.0059 (6)
C5B	0.0215 (7)	0.0221 (7)	0.0268 (7)	0.0017 (6)	0.0023 (6)	0.0051 (6)
C6B	0.0270 (8)	0.0208 (7)	0.0226 (7)	0.0041 (6)	0.0017 (6)	0.0002 (6)
C7B	0.0240 (7)	0.0253 (7)	0.0213 (7)	0.0047 (6)	0.0016 (5)	0.0001 (6)
N1A	0.0291 (7)	0.0246 (7)	0.0347 (7)	0.0050 (5)	0.0016 (6)	−0.0005 (6)
N2A	0.0241 (6)	0.0230 (6)	0.0229 (6)	0.0049 (5)	0.0032 (5)	0.0002 (5)
N3A	0.0258 (6)	0.0224 (6)	0.0241 (6)	0.0041 (5)	0.0025 (5)	−0.0028 (5)
C1A	0.0273 (8)	0.0256 (8)	0.0277 (8)	0.0078 (6)	0.0050 (6)	−0.0019 (6)
C2A	0.0262 (8)	0.0311 (8)	0.0354 (9)	0.0045 (6)	0.0070 (6)	0.0059 (7)
C3A	0.0293 (8)	0.0308 (8)	0.0286 (8)	0.0093 (6)	0.0093 (6)	0.0013 (6)
C4A	0.0268 (8)	0.0245 (7)	0.0248 (7)	0.0007 (6)	0.0048 (6)	−0.0001 (6)
C5A	0.0237 (7)	0.0293 (8)	0.0258 (8)	0.0029 (6)	0.0054 (6)	−0.0020 (6)
C6A	0.0301 (8)	0.0301 (8)	0.0256 (8)	0.0124 (6)	0.0060 (6)	0.0017 (6)

### Geometric parameters (Å, º)

**Table:**

O1B—C1B	1.2803 (18)	N1A—C2A	1.375 (2)
O2B—H2B	0.8400	N2A—C1A	1.3472 (19)
O2B—C7B	1.3019 (18)	N2A—C3A	1.3748 (19)
O3B—C7B	1.2249 (18)	N2A—C4A	1.4660 (19)
O4B—N2B	1.2303 (18)	N3A—H3AA	0.9100
O5B—N2B	1.2261 (18)	N3A—H3AB	0.9100
O6B—N1B	1.2300 (18)	N3A—H3AC	0.9100
O7B—N1B	1.2224 (18)	N3A—C6A	1.4844 (19)
N1B—C6B	1.4629 (19)	C1A—H1A	0.9500
N2B—C4B	1.4540 (18)	C2A—H2A	0.9500
C1B—C2B	1.441 (2)	C2A—C3A	1.352 (2)
C1B—C6B	1.433 (2)	C3A—H3A	0.9500
C2B—C3B	1.373 (2)	C4A—H4AA	0.9900
C2B—C7B	1.498 (2)	C4A—H4AB	0.9900
C3B—H3B	0.9500	C4A—C5A	1.517 (2)
C3B—C4B	1.385 (2)	C5A—H5AA	0.9900
C4B—C5B	1.381 (2)	C5A—H5AB	0.9900
C5B—H5B	0.9500	C5A—C6A	1.510 (2)
C5B—C6B	1.377 (2)	C6A—H6AA	0.9900
N1A—C1A	1.320 (2)	C6A—H6AB	0.9900
			
C7B—O2B—H2B	109.5	H3AA—N3A—H3AC	109.5
O6B—N1B—C6B	117.54 (13)	H3AB—N3A—H3AC	109.5
O7B—N1B—O6B	123.30 (14)	C6A—N3A—H3AA	109.5
O7B—N1B—C6B	119.17 (13)	C6A—N3A—H3AB	109.5
O4B—N2B—C4B	118.05 (13)	C6A—N3A—H3AC	109.5
O5B—N2B—O4B	123.43 (13)	N1A—C1A—N2A	111.69 (13)
O5B—N2B—C4B	118.52 (13)	N1A—C1A—H1A	124.2
O1B—C1B—C2B	120.31 (13)	N2A—C1A—H1A	124.2
O1B—C1B—C6B	124.78 (14)	N1A—C2A—H2A	124.8
C6B—C1B—C2B	114.84 (13)	C3A—C2A—N1A	110.33 (14)
C1B—C2B—C7B	119.59 (13)	C3A—C2A—H2A	124.8
C3B—C2B—C1B	121.69 (14)	N2A—C3A—H3A	127.0
C3B—C2B—C7B	118.70 (13)	C2A—C3A—N2A	105.94 (14)
C2B—C3B—H3B	120.0	C2A—C3A—H3A	127.0
C2B—C3B—C4B	120.03 (14)	N2A—C4A—H4AA	109.1
C4B—C3B—H3B	120.0	N2A—C4A—H4AB	109.1
C3B—C4B—N2B	119.02 (13)	N2A—C4A—C5A	112.48 (12)
C5B—C4B—N2B	119.37 (13)	H4AA—C4A—H4AB	107.8
C5B—C4B—C3B	121.60 (13)	C5A—C4A—H4AA	109.1
C4B—C5B—H5B	120.7	C5A—C4A—H4AB	109.1
C6B—C5B—C4B	118.69 (14)	C4A—C5A—H5AA	109.3
C6B—C5B—H5B	120.7	C4A—C5A—H5AB	109.3
C1B—C6B—N1B	120.14 (13)	H5AA—C5A—H5AB	108.0
C5B—C6B—N1B	116.69 (13)	C6A—C5A—C4A	111.50 (12)
C5B—C6B—C1B	123.12 (14)	C6A—C5A—H5AA	109.3
O2B—C7B—C2B	116.03 (13)	C6A—C5A—H5AB	109.3
O3B—C7B—O2B	121.99 (14)	N3A—C6A—C5A	112.37 (12)
O3B—C7B—C2B	121.96 (13)	N3A—C6A—H6AA	109.1
C1A—N1A—C2A	105.07 (13)	N3A—C6A—H6AB	109.1
C1A—N2A—C3A	106.97 (13)	C5A—C6A—H6AA	109.1
C1A—N2A—C4A	125.58 (13)	C5A—C6A—H6AB	109.1
C3A—N2A—C4A	127.43 (13)	H6AA—C6A—H6AB	107.9
H3AA—N3A—H3AB	109.5		
			
O1B—C1B—C2B—C3B	−178.23 (13)	C3B—C2B—C7B—O2B	179.26 (13)
O1B—C1B—C2B—C7B	0.3 (2)	C3B—C2B—C7B—O3B	1.0 (2)
O1B—C1B—C6B—N1B	−0.9 (2)	C3B—C4B—C5B—C6B	−0.6 (2)
O1B—C1B—C6B—C5B	176.43 (14)	C4B—C5B—C6B—N1B	178.87 (13)
O4B—N2B—C4B—C3B	3.8 (2)	C4B—C5B—C6B—C1B	1.5 (2)
O4B—N2B—C4B—C5B	−177.14 (13)	C6B—C1B—C2B—C3B	−0.9 (2)
O5B—N2B—C4B—C3B	−176.02 (14)	C6B—C1B—C2B—C7B	177.58 (12)
O5B—N2B—C4B—C5B	3.0 (2)	C7B—C2B—C3B—C4B	−176.73 (13)
O6B—N1B—C6B—C1B	154.04 (15)	N1A—C2A—C3A—N2A	0.13 (18)
O6B—N1B—C6B—C5B	−23.4 (2)	N2A—C4A—C5A—C6A	−69.71 (16)
O7B—N1B—C6B—C1B	−25.8 (2)	C1A—N1A—C2A—C3A	0.02 (18)
O7B—N1B—C6B—C5B	156.73 (14)	C1A—N2A—C3A—C2A	−0.23 (17)
N2B—C4B—C5B—C6B	−179.60 (13)	C1A—N2A—C4A—C5A	−80.85 (18)
C1B—C2B—C3B—C4B	1.8 (2)	C2A—N1A—C1A—N2A	−0.17 (18)
C1B—C2B—C7B—O2B	0.7 (2)	C3A—N2A—C1A—N1A	0.26 (18)
C1B—C2B—C7B—O3B	−177.60 (13)	C3A—N2A—C4A—C5A	97.42 (17)
C2B—C1B—C6B—N1B	−178.03 (12)	C4A—N2A—C1A—N1A	178.83 (13)
C2B—C1B—C6B—C5B	−0.7 (2)	C4A—N2A—C3A—C2A	−178.77 (14)
C2B—C3B—C4B—N2B	177.99 (13)	C4A—C5A—C6A—N3A	175.16 (12)
C2B—C3B—C4B—C5B	−1.0 (2)		

### Hydrogen-bond geometry (Å, º)

**Table:**

*D*—H···*A*	*D*—H	H···*A*	*D*···*A*	*D*—H···*A*
O2*B*—H2*B*···O1*B*	0.84	1.66	2.4484 (15)	155
N3*A*—H3*AA*···N1*A*^i^	0.91	1.92	2.7987 (19)	162
N3*A*—H3*AB*···O1*B*^ii^	0.91	2.03	2.8153 (17)	144
N3*A*—H3*AC*···O3*B*^iii^	0.91	2.07	2.9546 (17)	165
C4*A*—H4*AB*···O4*B*^iv^	0.99	2.53	3.3572 (19)	142

Symmetry codes: (i) −*x*, −*y*, −*z*; (ii) −*x*, −*y*+1, −*z*+1; (iii) *x*+1, *y*, *z*; (iv) −*x*+1, −*y*+1, −*z*.
